# Screening, identifying, and treating chronic kidney disease: why, who, when, how, and what?

**DOI:** 10.1186/s12882-024-03466-5

**Published:** 2024-01-25

**Authors:** Douglas R. Farrell, Joseph A. Vassalotti

**Affiliations:** 1 Department of Medicine, Division of Nephrology, Icahn School of Medicine at Mount Sinai, 10029 New York, NY USA; 2 National Kidney Foundation, Inc, New York, NY USA

**Keywords:** Albuminuria, CKD, Screening, Nephrology, Hypertension, Diabetes

## Abstract

1 in 7 American adults have chronic kidney disease (CKD); a disease that increases risk for CKD progression, cardiovascular events, and mortality. Currently, the US Preventative Services Task Force does not have a screening recommendation, though evidence suggests that screening can prevent progression and is cost-effective.

Populations at risk for CKD, such as those with hypertension, diabetes, and age greater than 50 years should be targeted for screening. CKD is diagnosed and risk stratified with estimated glomerular filtration rate utilizing serum creatinine and measuring urine albumin-to-creatinine ratio. Once identified, CKD is staged according to C-G-A classification, and managed with lifestyle modification, interdisciplinary care and the recently expanding repertoire of pharmacotherapy which includes angiotensin converting enzyme inhibitors or angiotensin-II receptor blockers, sodium-glucose-cotransporter-2 inhibitors, and mineralocorticorticoid receptor antagonists.

In this paper, we present the why, who, when, how, and what of CKD screening.

## Why?

Chronic kidney disease (CKD), defined as reduced glomerular filtration rate (GFR) and/or presence of albuminuria, is estimated to have a prevalence of over 850 million individuals worldwide [1]. In the United States, 1 in 7 (approximately 14%) of the adult population are estimated to have CKD. Patients with CKD experience higher levels of morbidity and mortality compared to the general population and cost the healthcare system a disproportionate amount [2]. Despite its prevalence and morbidity, as little as 9% of patients with CKD are aware of their condition [3]. Given this high prevalence, simplicity of detection, and numerous available therapeutic options to delay its progression; major clinical practice guideline organizations in nephrology and diabetes recommend CKD screening and have called upon the United States Preventive Services Taskforce (USPSTF) to prioritize reexamining recommendations for CKD screening [4, 5]. We believe CKD fulfills the WHO principles for important disease screening because it confers large morbidity with a latent asymptomatic stage, has ubiquitous and inexpensive tests for identification, and has numerous therapeutic options for its treatment [6]. Early treatment of CKD not only can prevent the progression to dialysis but can also reduce cardiovascular events.

## Who and when?

While a strategy of general population or mass screening for all maximizes the detection of CKD, it is not necessarily cost-effective. Thus, identifying high-risk populations is prudent to increase screening yield and cost effectiveness. Traditionally, the health economic literature has utilized the arbitrary threshold of $50,000 for each increase in quality-adjusted life year (QALY) gained as the standard for acceptable value-based care [7]. In the past decade, this threshold has been controversial, with current literature and analyses using $100,000-150,000/QALY as likely high value [8, 9]. A landmark 2003 paper suggested that yearly dipstick screening for proteinuria followed by medical management utilizing angiotensin converting enzyme inhibitors (ACEi) or angiotensin-II receptor blockers (ARB) for patients without hypertension (HTN) and diabetes mellitus (DM) could cost as much as $282,000/QALY, though limiting screening to those with HTN may only cost $18,000/QALY [10]. This analysis underestimated the benefits of screening, because the impact of sodium-glucose co-transporter-2 inhibitor (SGLT2i) therapy was not considered. As mentioned, the prevalence of CKD in the adult population of the United States is estimated to be approximately 14%, but this prevalence of CKD increases up to 40% in patients with diabetes, 33% in seniors age 65 years and older, and 22% in those with hypertension [2]. One study found that selecting a cohort of patients with either diabetes, hypertension, or age at least 50 years resulted in a CKD prevalence of 34% [11]. 

### Diabetes

**Fig. 1:**
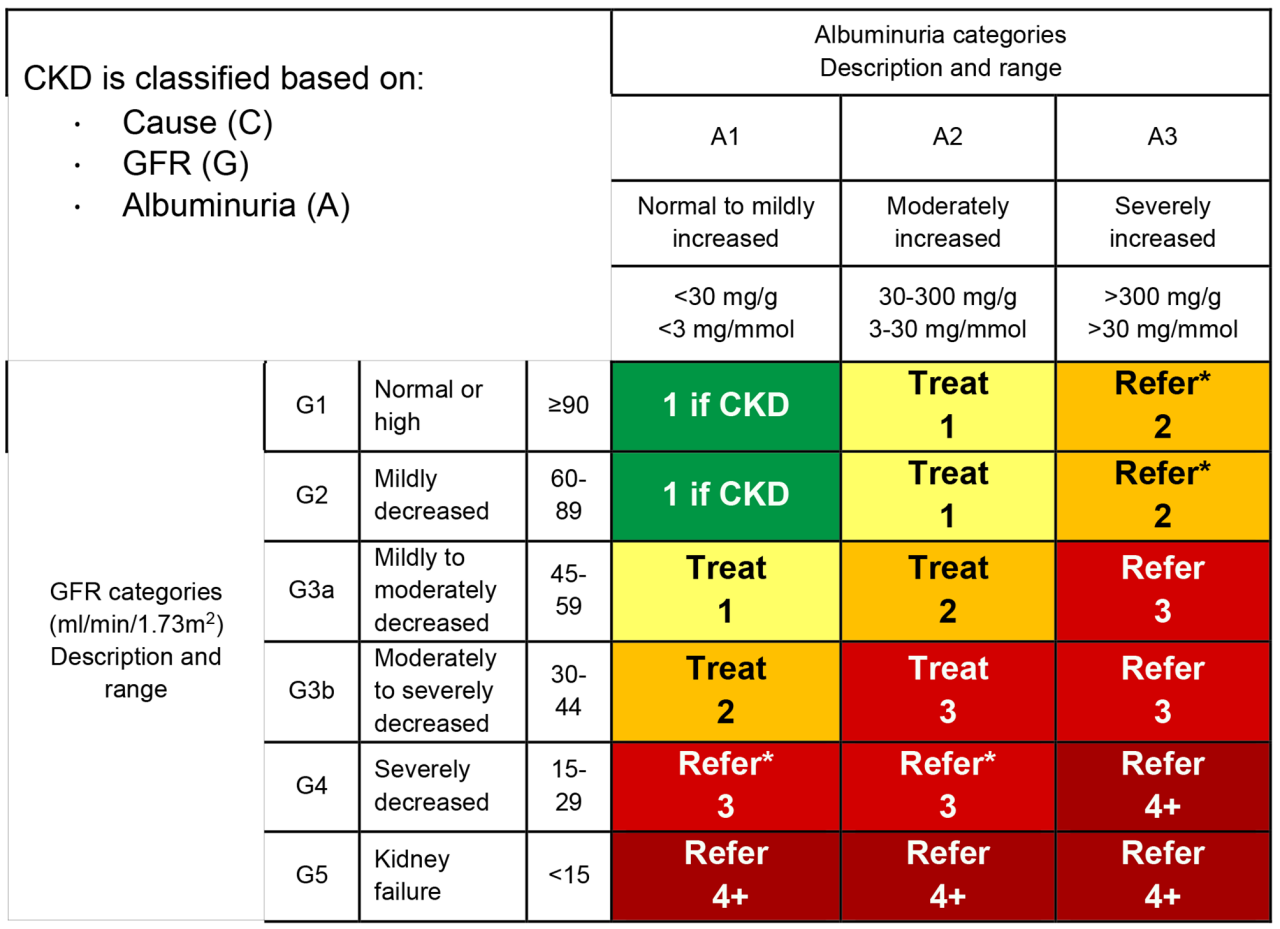
Heat map that reflects prognosis, frequency of visits and indications for referral. Classification heat map that reflects prognosis, recommended frequency of visits and indications for nephrology referral. The GFR and albuminuria grid depict the risk of progression, morbidity and mortality by color from best to worst (green, yellow, orange, red, deep red). The numbers in the boxes are a guide to the frequency of visits (number of times per year). Green can reflect CKD with normal eGFR and ACR only in the presence of other markers of kidney damage, such as imaging showing polycystic kidney disease or kidney biopsy abnormalities, with follow-up measurements annually; yellow requires caution and measurements at least once per year; orange requires measurements twice per year; red requires measurements at 3 and deep red 4 times per year. These are general parameters only based on expert opinion and must take into account underlying comorbid conditions and disease state, as well as the likelihood of impacting a change in management for any individual patient. Refer indicates nephrology services are recommended. *Referring clinicians may wish to discuss with their nephrology service depending on local arrangements regarding treating or referring. This figure is adapted from KDIGO [29]

Diabetes is the most common cause of CKD, incident and prevalent end-staged kidney disease (ESKD). Moreover, the prevalence of type-2 diabetes in the adult population continues to rise, driven by population aging and the obesity epidemic [12, 13]. As previously noted, up to 40% of patients with diabetes have detectable CKD by estimated glomerular filtration rate (eGFR) and/or urine albumin-creatinine ratio (uACR). Three- and five-year incidence of new moderately elevated albuminuria (CKD A2 historically termed microalbuminuria (see Fig. 1) )in those with previously normal urine studies have been predicted to be 12.8% and 23.9%, respectively [14]. Given our abilities to reduce the rate of progression of diabetic kidney disease, recommendations for yearly CKD screening for diabetic patients have been in place for almost 20 years. Studies looking at the impact of yearly screening overwhelmingly show cost-effectiveness, in addition to decreasing ESKD incidence by up to 40% [15–18]. Despite consensus among clinical practice guideline recommendations for yearly screening, less than 40% of diabetic patients receive necessary screening tests [2, 14, 19, 20]. Along with guidelines, we strongly recommend yearly CKD screening for patients with diabetes. Screening for type 2 diabetes (T2DM) should occur at time of diagnosis while type 1 diabetes (T1DM) should start five years after diagnosis; this difference is due to the rapid onset of presentation in T1DM and frequently delayed diagnosis of T2DM [21].

### Hypertension

Hypertension is one of the most common causes or complications of CKD and ESKD. When ESKD incidence was studied in over 300,000 adults without CKD, even patients with high-normal blood pressure (BP) (systolic BP 120–129 mm Hg) had an incident ESKD age-adjusted relative risk (RR) of 1.62 per 100,000 person-years when compared to patients with normal BP defined as systolic pressure less than 120 mm Hg [22]. More than 20% of patients with HTN have evidence of CKD [2]. For the HTN population without evidence of albuminuria, 3- and 5-year albuminuria incident rate was predicted to be 14.8% and 21.7% respectively [14]. An economic analysis revealed yearly screening for CKD followed by treatment in the hypertensive population has been found to be cost-effective [10, 23]. We recommend yearly screening for CKD in patients with hypertension.

### Age

Age is a risk factor for CKD in part because normal aging physiology results in decreases in GFR, particularly after age 50 years, leading to controversy in the nephrology literature regarding absolute or age-adjusted cutoffs for CKD detection and risk stratification. By the decades 60–69 and 70–79 years, the CKD prevalence increases to 20% and 42% respectively [2, 24, 25]. Boulware et al. found for those without hypertension and diabetes, yearly screening for CKD starting at age 60 was a deflection point in cost-effectiveness [10]. In another study by Hoerger et al., screening patients without hypertension and diabetes starting at age 50 did not become cost-effective until the interval of screening was increased to every 5–10 years. When looking specifically at age, targeting age over 50 years was the most cost-effective [23]. A recently published study reexamined screening of the general population in the era of SGLT2i, and found cost-effectiveness starting as early as age 35, though this did not analyze the effect of comorbidities [26]. In our opinion, screening patients for CKD without associated comorbidities is reasonable to begin after age 50, though there is not enough data to inform testing frequency. In the U.S., seniors and Medicare eligibility are defined at age 65 years, making this an attractive alternative threshold.

### Cardiovascular disease

Cardiovascular disease (CVD) is a broad category in the literature that includes heart failure (HF), coronary artery disease (CAD), and acute myocardial infarction (AMI). The presence of cardiovascular disease increases the prevalence of CKD from ~ 12% to nearly 40% [2]. Though, when adjusted for presence of hypertension and diabetes, CVD alone does not capture the same proportion of CKD prevalence [11]. Regardless, CKD and CVD are closely interrelated and thus screening should be extended to all patients with underlying cardiovascular disease.

### Social determinants of health

Kidney disease disproportionately affects individuals of lower socioeconomic status. CKD prevalence inversely correlates with education level and yearly income [27]. Individuals living in more socioeconomically deprived areas have a higher incidence of diabetes, hypertension, and ESKD. Unfortunately, those with social deprivation were less likely to have access to pre-emptive kidney transplant and home dialysis (patient-centric kidney failure replacement therapies). A significantly greater percentage of Black and Hispanic individuals were more likely to live in socioeconomically deprived areas compared to white individuals and have increased prevalence of CKD [27, 28]. Given disparities in kidney disease and outcomes, additional attention to screening individuals with socioeconomic disadvantage for CKD promotes health equity, and thus we recommend screening for this population.

See Table 1 for a comprehensive list of risk conditions for CKD.

**Table 1:** CKD risk conditions

Diabetes
Hypertension
Cardiovascular disease
Acute kidney injury
Family history of kidney disease and/or genetic kidney disorders
Obesity
Social deprivation
Age 50 years or older*
Urinary tract obstruction
Recurrent kidney stones
Systemic infection (i.e., HIV and viral hepatitides)
Malignancy
Autoimmune disorders
Low birth weight

*Age thresholds have varied in the literature including 50, 55, 60 and 65 years. In the U.S., seniors and Medicare eligibility are defined at age 65 years, making this an attractive threshold

## How?

CKD is defined by either a glomerular filtration rate (GFR) less than 60 ml/min/1.73m^2^ and/or evidence of kidney damage, usually defined by the presence of albuminuria, for 3 or more months. Other markers of kidney damage include urine sediment abnormalities, structural abnormalities on imaging, and kidney biopsy morphologic findings. Once identified, CKD is risk-stratified by cause-GFR category-albuminuria category (C-G-A classification), see the heat map Fig. 1 [29].

### GFR

GFR is considered the standard assessment of kidney function and has been assessed in routine clinical practice with eGFR using the endogenous filtration marker serum creatinine [30]. For screening purposes, we recommend testing serum creatinine which is included on the basic and comprehensive metabolic panels (BMP and CMP, respectively). Equations for estimating GFR using creatinine have undergone multiple iterations for improvement in precision and reduction in bias. The eGFR should be calculated utilizing the most recent 2021 CKD-EPI creatinine equation that was refit without race as a coefficient in the U.S [31, 32]. Clinicians may utilize the NKF website for the eGFR calculator [33]. 

**Fig. 2:**
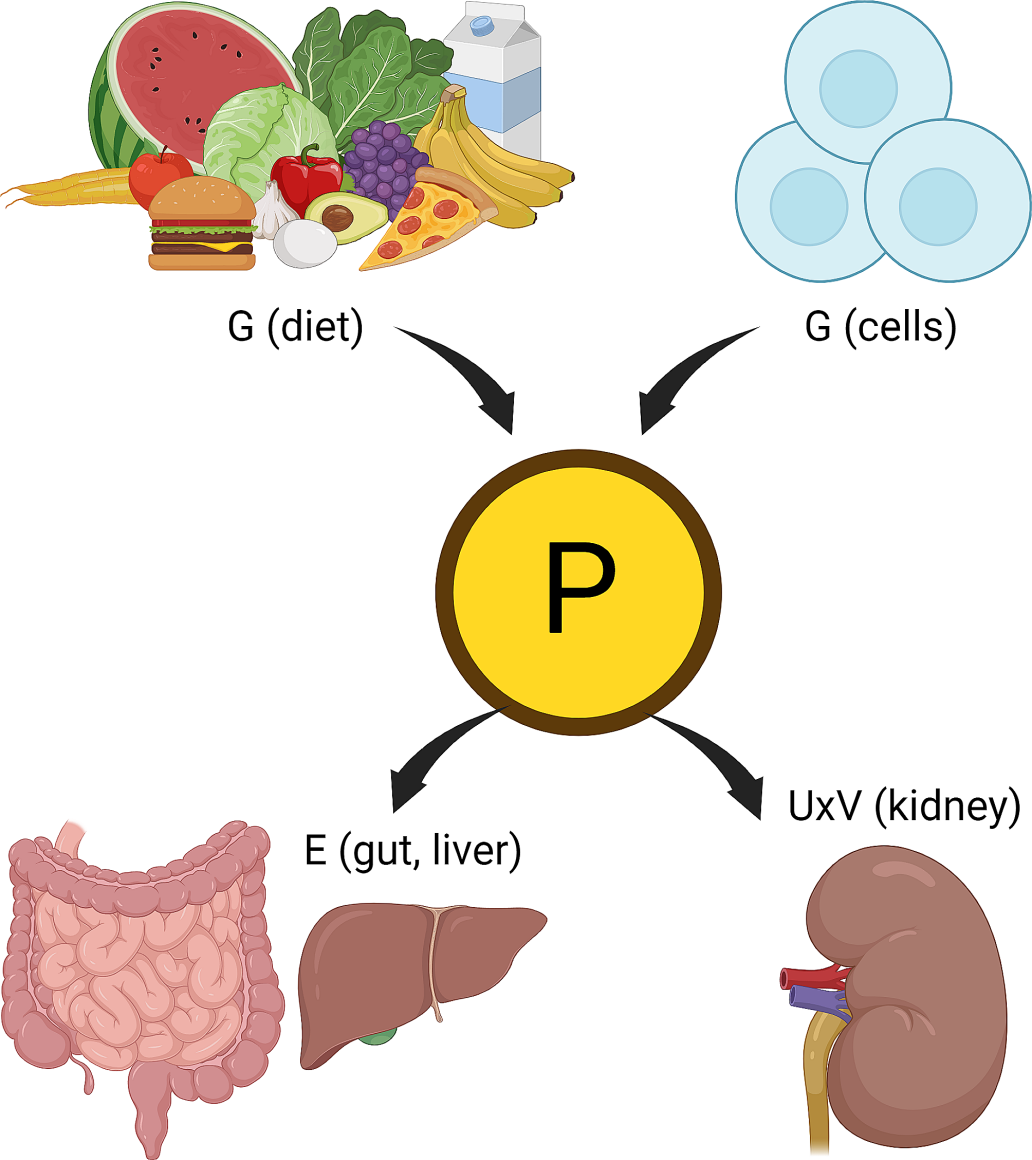
Conceptual depiction of GFR and non-GFR determinants of plasma (P) biomarker. Non-GFR determinants include generation (G), and non-renal elimination (E), tubular secretion and tubular reabsorption (both not labeled). GFR determinants include urine concentration of P (U) and urine volume (V). Adapted from Stevens LA et al. Created with BioRender.com. [34]

Creatinine, while the current clinical standard endogenous biomarker for estimating GFR, has numerous non-GFR determinants that may over- and under-estimate GFR, see Table 2; Fig. 2 [34, 35]. Cystatin-C has been under investigation as an endogenous biomarker for estimating GFR that does not share the same limitations of creatinine, and when combined with creatinine results in a more accurate estimation [31]. At this time, given limited clinical availability of cystatin-C, we recommend addition of cystatin-C by utilizing the 2021 CKD-EPI combined creatinine and cystatin-C equation when more precision is required in the absence of non-GFR determinants for either biomarker.

**Table 2:** Non-GFR determinants of blood creatinine and cystatin C concentrations

	Non-GFR Determinants	
	Creatinine	Cystatin C
GFR overestimation due to decreased creatinine or cystatin C	Physiologic factors:	Physiologic factors:
	unknown	unknown
	**Pathologic conditions**:	**Pathologic conditions**:
	amputation, frailty, anorexia, sarcopenia, liver cirrhosis, thyroid disease, chronic illness, critical illness; extra-renal elimination e.g.intestinal bacterial metabolism, spinal cord injury and progressive neuromuscular disease	thyroid disease
	**Diet**:	**Diet**:
	vegan diet	unknown
**GFR underestimation due to increased creatinine or cystatin C**	**Physiologic factors**:	**Physiologic factors**:
	high muscle mass e.g. bodybuilders	smoking, lower lean body mass
	**Pathologic conditions**:	**Pathologic conditions**:
	obesity, rhabdomyolysis, thyroid disease	Obesity, diabetes, inflammation, thyroid disease, hypercortisolism
	**Diet**:	**Diet**:
	high meat consumption, creatine supplements	unknown
	**Drugs**:	**Drugs**:
	Inhibition of tubular secretion- trimethoprim, cobicistat, dolutegravir, fenofibrate, olaparib, ritonavir, cimetidine	glucocorticoids

Adapted with permission of Pierre CC et al. [35]

The serum creatinine and cystatin-C are currently the standard filtration markers for estimating GFR, but still may exhibit significant individualized variability from the true GFR. In the presence of non-GFR determinants for creatinine, the CKD-EPI 2012 eGFR equation using cystatin-C only may be the best assessment of kidney function. When absolute accuracy of GFR is necessary such as safety of kidney donation or critical medication administration (such as chemotherapy), GFR can be directly measured (mGFR). Direct measurement of GFR generally includes administering exogenous markers, such as the iohexol and iothalmate, and measuring its clearance from serum, since inulin is no longer available [34]. 

Lastly, eGFR is traditionally indexed to a body surface area (BSA) of 1.73 m^2^ for standardization of measurement across size variability. For patients at extremes of BSA, indexing GFR may lead to large discrepancies from true GFR, and thus de-indexing may be appropriate [36]. 

### Albuminuria

Under ordinary conditions, the glomerular basement membrane limits the passage of large molecules such as albumin from filtering into the urinary space. The albumin that is filtered, is subsequently reabsorbed in the proximal tubule. Thus, the presence of albumin generally suggests damage to the filtration barrier or proximal tubule [37]. Total proteins, measured in the urine using 3 different assays, represent a broad array of proteins including normally excreted tubular proteins such as uromodulin (“Tamm-Horsfall protein”), whereas albumin is thought to be more specific to the presence of nephron dysfunction. Significant albuminuria (defined as 30 mg or more of urinary albumin excretion per gram of creatinine on a random specimen) is an independent risk factor for ESKD, acute kidney injury (AKI), GFR loss, cardiovascular events, as well as cardiovascular and all-cause mortality [38–41]. Cardiovascular risk with albuminuria is a continuous variable that has been demonstrated with albumin excretion less than the recommended threshold of 30 mg/day [42, 43]. 

The former gold-standard for detection and quantification of albuminuria is a 24-hour urine collection, which is cumbersome and subject to over and under collection. Instead, measuring a spot uACR correlates well with the 24-hour excretion and is currently recommended for routine practice [44–47]. Urinary albumin concentration can be increased by physical activity, fever, stress, metabolic perturbations, and other conditions, making first-morning samples optimal [48, 49]. Given the variability in albumin excretion, confirming elevated uACR with repeat testing should be emphasized [29, 50–52]. The uACR is in the process of being standardized.

While quantitative tests for albuminuria are standard, semi-quantitative approaches have been studied because they may reduce costs, expand access to clinics without laboratory services, and are point-of-care which may allow for immediate identification for clinical counseling and treatment decisions. When studied, semi-quantitative tests have shown reasonable sensitivity, but do not match the quantitative uACR [53, 54]. Given their potential benefits for expansion of access, semi-quantitative is a reasonable screening test in the absence of quantitative uACR availability. Semiquantitative or qualitative screening tests should be positive in > 85% of individuals with A2 or moderately increased albuminuria to be useful for patient screening [55]. A recent international CKD screening review addressed differing approaches to screening versus case finding in low-income, middle-income, and high-income countries with considerations including less expensive urine protein dipstick testing in low-income countries versus a broader population screened with eGFR and uACR and increased interval of screening in high-income countries [56]. Highly motivated and high socioeconomic status individuals will likely prefer additional screening tests with increased frequency, but the benefits of this approach have not been clearly demonstrated. The focus of this review is the impact of the minimum standard screening to overcome potential inequities in CKD care that may be driven by lower uACR testing among individuals with risk conditions and low neighborhood income and low education level that were shown in a recent U.S. study of over 7 million adults with diabetes [20]. See Table 3 for a comparison of the various tests for urinary proteins [35]. 

**Table 3:** KDIGO albuminuria classification with associated measurements of urine proteins

Terms	Albuminuria Category	Albumin (mg/ 24 h urine)	uACR (mg/g)	uPCR (mg/g)	Dipstick Proteinuria
Normal to mildly increased	A1	< 30	< 30	< 150	Negative to trace
Moderately increased	A2	30–300	30–300	150–650	Trace to 1+
Severely increased	A3	> 300	> 300	> 650	+ 2 or greater
Nephrotic range	A3 Nephrotic range	> 2000	> 2000	> 3500	+ 2 or greater

KDIGO urine albumin to creatinine ratio (uACR) classifications with corresponding 24-hour urine albumin concentrations, uACR measurements, and terms. Corresponding urine protein to creatinine ratio (uPCR) and dipstick protein results using approximate conversions are also shown in the last two columns. Adapted from Pierre C et al. [35]

## What?

### Confirmation and staging

The diagnosis of CKD requires demonstration of reduced GFR or evidence of kidney damage for 3 or more months. Investigation for chronicity includes trending prior laboratory results for confirmation of stability. In the absence of previous eGFR results, observation of small echogenic kidneys on imaging, or imaging manifestations of secondary hyperparathyroidism confirm chronicity. If duration of kidney disease is unable to be confirmed, AKI should be ruled out. Once identified, CKD should be staged based on the cause-GFR-albuminuria (C-G-A) classification [29]. Optimal care of patients with CKD includes a multifaceted approach with optimization of diet, lifestyle, comorbidities, and pharmacotherapy.

### Comorbidities

#### Hypertension

For patients with CKD and hypertension, clinical practice guidelines recommend intensive BP control goal of < 130/80 mm Hg in most patients and < 120/80 mm Hg for high-risk individuals [57, 58]. For the general population, intensive hypertension control has been shown to have cardiovascular and mortality benefits in large, randomized controlled trials (RCT) and meta-analysis [59, 60]. Application to CKD patients was initially conflicting, as many trials did not include significant kidney disease. Subgroup analysis of the SPRINT trial suggested that there may be increased CKD incidence with intensive BP control [61]. Meta-analysis data suggests that intensive BP control may prevent progression of kidney disease in those with proteinuric CKD [62]. Cardiovascular and mortality benefits seen in SPRINT and meta-analysis data rather than kidney-specific protection are the primary benefits for CKD patients with intensive BP targets [60, 63–66]. 

Measuring blood pressure is a routine procedure in outpatient and inpatient practice that is often performed inaccurately. In an office setting, blood pressure should be measured with an appropriate fitting cuff on a patient’s bare upper arm rested at the level of the heart after at least five minutes of rest. Other accepted forms of measurement include 24-hour ambulatory blood pressure monitoring (ABPM) and home blood pressure monitoring (HBPM), which provide more accurate representation of the patient’s blood pressure outside of an office environment [67]. Particularly when targeting systolic blood pressure in the range of 110 to 130 mm Hg, assessing blood pressure outside of the clinic to provide additional datapoints in between visits using a validated and calibrated HBPM or when available in select cases ABPM is important for monitoring and safety.

#### Diabetes Mellitus

For patients with diabetes and CKD, tight glycemic control alone has been shown to prevent the progression of diabetic kidney disease [68–73]. Hemoglobin-A1c targets of < 7% are generally accepted as goal for reducing microvascular complications. This benefit must be outweighed by the potential for increased adverse events associated with intensive control [21]. 

### Pharmacotherapy

#### GFR adjustments

As many as 50% of all FDA approved medications are renally excreted, and alterations in excretion may lead to systemic toxicities [74]. For patients with CKD, medication regimens should be evaluated and dosed appropriately according to the patient’s eGFR [29, 51]. The level of kidney function should guide prescription practice including avoidance or dose adjustment of medications.

#### ACEi or ARB

For the previous decades, ACEi and ARBs have been the mainstay of therapy for slowing the progression of proteinuric kidney disease. Numerous trials aggregated in meta-analysis have shown that both ACEi and ARBs reduce eGFR decline and progression to ESKD in both diabetic and non-diabetic proteinuric kidney disease (A3 or uPCR > 650 mg/g, uACR > 300 mg/g) [75–79]. These medications should generally be titrated to their maximally tolerated doses.

Patients with diabetes and moderate albuminuria (A2 or uACR 30-300 mg/g) benefit from maximally tolerated ACEi or ARB given trial data exhibiting decreased development of increasing albuminuria to A3 [80, 81]. For non-diabetic patients with moderate albuminuria, less data exists for benefit of ACEi or ARBs, but patients still likely derive benefit from albuminuria reduction with these agents [82]. ACEi and ARBs do not to have the same eGFR preservation in patients with non-proteinuric kidney disease, and thus are not recommended for this subgroup [77]. 

#### SGLT2i

SGLT2is initially started as glucose lowering agents for T2DM but were subsequently shown to have significant cardiovascular and kidney protection in large high quality RCTs [83–85]. In a meta-analysis, SGLT2is even have kidney protective effects for diabetic patients without albuminuria [86]. Thus, for patients with diabetes and evidence of CKD (eGFR > 20), SGLT2is are strongly recommended after addition of maximally tolerated ACEi or ARB.

For patients with non-diabetic kidney disease, SGLT2is were assessed in two large kidney outcome RCTs, DAPA-CKD and EMPA-KIDNEY. For non-diabetic patients with A3, SGLT2i exhibited similar kidney, cardiovascular, and mortality benefits as previous trials in patients with diabetes. Evidence for those with moderate-to-no albuminuria is limited to modest GFR decline attenuation, though does not appear to have significant benefit on the primary outcomes [87, 88]. Overall, for patients with non-diabetic kidney disease (eGFR > 20) and albuminuria, SGLT2i are also strongly recommended after addition of maximally tolerated ACEi or ARB.

#### MRA

The steroidal mineralocorticoid receptor antagonist (MRA) spironolactone has been shown to attenuate albuminuria in diabetic kidney disease without outcomes data but had numerous disadvantages including sexual side effects and significant hyperkalemia that can be prolonged for as long as 1 week after discontinuation as a result of long-acting metabolites such as canrenone [89, 90]. That noted, spironolactone is evidenced-based for the treatment of heart failure with reduced ejection fraction (HFrEF) based on the RALES trial and is also an excellent choice for resistant HTN in CKD [91]. Lastly, spironolactone is generally used for patients with cirrhosis and ascites. Eplerenone is an additional steroidal MRA used for patients who cannot tolerate spironolactone. More recently, the selective non-steroidal mineralocorticoid receptor antagonist finerenone has been of interest given the activity of this receptor in diabetic kidney disease. In two large RCTs, finerenone has demonstrated significant cardiovascular and kidney protection for patients with proteinuric kidney disease due to type-2 diabetes mellitus when used with maximally tolerated ACEi or ARB. An additional benefit of finerenone, is that it exhibited lower hyperkalemic events in addition to no sexual side-effects observed with other less-selective MRAs. Importantly, finerenone should be avoided in those with decompensated cirrhosis [92–94] There are no head-to-head trials comparing spironolactone, eplerenone, and finerenone. Thus, for patients with proteinuric diabetic kidney disease (T2DM only), finerenone is recommended for both eGFR preservation and cardiovascular protection. Because finerenone is used in CKD with maximally dosed ACEi or ARB, potassium monitoring is an important safety consideration and many patients with reduced eGFR will require concomitant potassium binder therapy.

#### HMG-CoA reductase inhibitors (statins)

CKD is a well-known risk for cardiovascular disease and CVD mortality [95, 96]. It has been recognized by both nephrology and cardiovascular organizations as one of the highest risk factors for the development of CVD [97]. The Study of Heart and Renal Protection (SHARP) trial showed that the addition of simvastatin 20 mg and ezetimibe 10 mg for CVD primary prevention significantly reduced major atherosclerotic events in patients with non-dialysis dependent CKD age 40 or greater by nearly 22%. The results of the SHARP trial have been further supported by multiple meta-analyses suggesting that patients with CKD derive statistically significant reduction in cardiovascular events with HMG-CoA reductase inhibitor (statin) treatment [98–101]. CVD risk attenuation of statins is the result of reduction in low-density lipoprotein cholesterol (LDL-c), rather than a class-dependent effect [102]. Guidelines vary in their lipid-pharmacotherapy recommendation. KDIGO and KDOQI agree that statins should be initiated in all patients aged 50 years and older with CKD, in addition to patients younger than 50 years with > 10% cardiovascular event 10-year risk [103, 104]. The AHA/ACC acknowledges CKD as a significant risk factor for CVD, but ultimately recommend utilizing statins for target LDL-c reduction of > 50% for high-risk patients (> 20% ASCVD risk) and 30–49% for intermediate risk (7.5–20% ASCVD risk). ESC/EAS guidelines are more aggressive and recommend LDL-c targets of < 70 mg/dL (1.8mmol/L) for patients with GFR 30–59, and < 55 mg/dL (1.4 mmol/L) for patients with GFR < 30. Given recent trends in CVD, a new risk score titled PREVENT has been published by the AHA that incorporates kidney disease to more accurately predict 10- and 30- year risk of cardiovascular disease [105]. 

Multiple RCTs including the SHARP trial show that patients on dialysis do not exhibit the same significant reduction cardiovascular events, which may be explained by the fact that patients on dialysis are more likely to have sudden cardiac death [101, 106, 107]. 

Data from the SHARP trial also suggests statins do not increase risk of hepatitis for patients with CKD compared to placebo, making routine monitoring for liver enzymes unnecessary [101]. CKD increases the risk of statin-related myopathy, but in the SHARP trial this risk was minimal. Risk of statin-induced rhabdomyolysis appears to be highest with the use of simvastatin above doses of 40 mg, or when statins are used with known CYP3A4 drug-interactions [108]. Rosuvastatin doses above 10 mg should be avoided in patients with eGFR < 30 given its association with hematuria, proteinuria, and progression to dialysis in this group [109]. Lastly, for patients with statin-intolerance or adverse events, a referral to a lipid specialist should be considered for advanced therapies including PCSK-9 inhibitors.

### Referrals

#### Medical Nutrition Therapy

Patients with CKD are at higher risk of malnutrition and are likely to have risk factors that benefit from specific dietary modifications such as sodium, calorie, and carbohydrate limited diets. It is recommended that patients at all stages of CKD be referred to medical nutrition therapy (MNT), which can be performed by registered dieticians or diabetes educators [110]. Individualized dietary counseling or MNT should routinely be part of the care plan so that patients can make better informed choices to maximize their long-term health. Nutrition interventions have demonstrated improvements in glucose and blood pressure control, slowing of CKD progression, and delaying need for dialysis [111]. 

#### Pharmacist

Inequities in SGLT2is have been demonstrated in practice, and probably also exist for finerenone [112]. Pharmacists may be the health care professionals best able to help patients select the medication choices and resources that allow the least out of pocket costs to optimize access. In addition, complex medication management and medication reconciliation may be improved with pharmacist engagement. Pharmacist interventions have been shown to effectively improve glycemic control and blood pressure, both crucial for patients with CKD [113, 114]. Data specifically for CKD patients is limited and focuses more on dialysis patients but does suggest benefit to management of the numerous risk factors and complications of CKD [115, 116]. Thus, the addition of a pharmacist to the care team may be an important intervention for access to medications and health equity.

#### Nephrologist

**Fig. 3:**
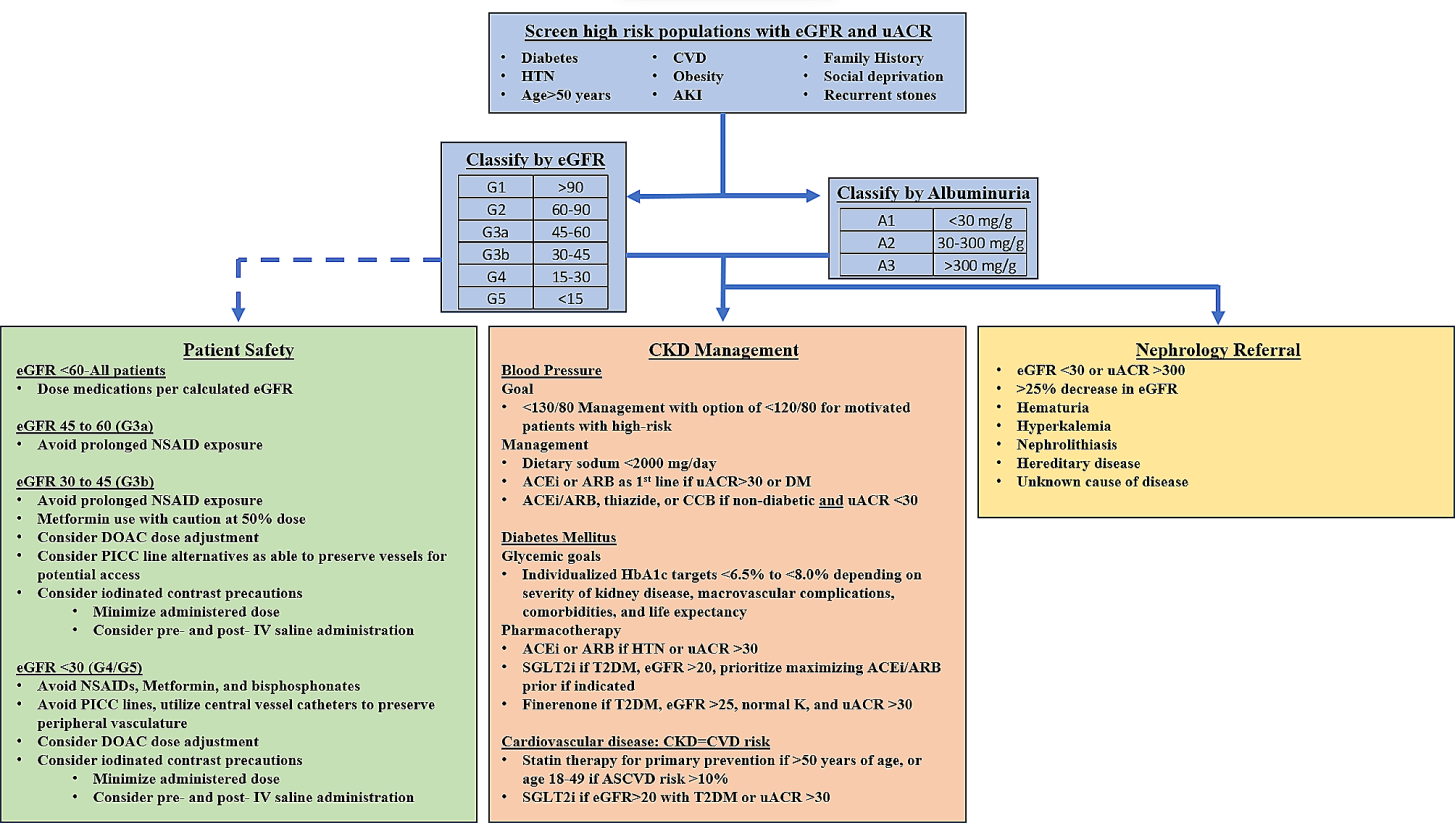
Summative diagram for CKD screening, staging, and management. Adapted from Vassalotti et al. [119]

Referral to a nephrologist is an important consideration for the patient with CKD. In addition to implementation of evidence-based pharmacotherapy, nephrologists are also well-versed in the screening for and management of the many complications of advanced kidney disease including anemia, metabolic bone disease, as well as dialysis and transplant planning. Early referral to a nephrologist (defined by timing until dialysis) has been associated with mortality benefit based on meta-analysis data from about 100 observational studies and one RCT [117, 118]. KDIGO guidelines for CKD management recommend opinion-based criteria for nephrology referral including AKI, unknown cause of disease, rapidly progressive CKD, eGFR < 30 ml/min/1.73 m^2^, persistent A3 (> 300 mg/g), unexplained hematuria, refractory hypertension, persistent hyperkalemia, recurrent nephrolithiasis, and hereditary kidney disease [29, 51]. Please refer to Fig. 3 for a summative figure of the who, how, and what of CKD screening [119]. 

## Conclusion

CKD is a disease in which screening efforts will allow initiation of therapy shown to have significant impact on progression, cardiovascular risk reduction and mortality while maintaining cost-effectiveness. Patients at elevated risk for CKD, including patients with diabetes, hypertension, and age greater than 50 years should be screened by calculating eGFR alongside uACR measurement. Once CKD is identified, patients should be stratified utilizing the C-G-A classification. Management of CKD includes strict blood pressure and glycemic control, eGFR appropriate adjustment of pharmacotherapy regimen, MNT referral, and consideration for initiation of statins, ACEi or ARB, SGLT2i, and MRA. Early referral to a nephrologist for patients with high-risk of progression has been shown to improve outcomes and is recommended.

## Data Availability

Not applicable.

## Abbreviations

AKI: Acute kidney injury
AMI: Acute myocardial infarction
ABPM: Ambulatory blood pressure monitoring
ACEi: Angiotensin converting enzyme inhibitors
ARB: Angiotensin-II receptor blockers
BMP: Basic metabolic panel
BP: Blood pressure
BSA: Body surface area
C-G-A: Cause-GFR-albuminuria
CVD: Cardiovascular disease
CKD: Chronic Kidney Disease
CMP: Comprehensive metabolic panel
CAD: Coronary artery disease
DM: Diabetes mellitus
ESKD: End stage kidney disease
eGFR: Estimated glomerular filtration rate
GFR: Glomerular filtration rate
HF: Heart failure
HFrEF: Heart failure with reduced ejection fraction
HBPM: Home blood pressure monitoring
HTN: Hypertension
LDL-c: Low density lipoprotein cholesterol
mGFR: Measured glomerular filtration rate
MNT: Medical nutrition therapy
MRA: Mineralocorticoid receptor antagonist
QALY: Quality-adjusted life year
RCT: Randomized control trial
RR: Relative risk
SGLT2i: Sodium-glucose co-transporter-2 inhibitor
T1DM: Type 1 diabetes
T2DM: Type 2 diabetes
USPSTF: United States Preventative Services Taskforce
uACR: Urine albumin-creatinine ratio
uPCR: Urine protein-creatinine ratio
